# Health-related characteristics and preferred methods of receiving health education according to dominant language among Latinos Aged 25 to 64 in a large Northern California health plan

**DOI:** 10.1186/1471-2458-8-305

**Published:** 2008-09-09

**Authors:** Nancy P Gordon, Carlos Iribarren

**Affiliations:** 1 Division of Research, Kaiser Permanente Medical Care Program, 2000 Broadway, Oakland, CA 94612, USA

## Abstract

**Background:**

Latinos are a fast growing segment of the U.S. health care population. Acculturation factors, including English fluency, result in an ethnic group heterogeneous with regard to SES, health practices, and health education needs. This study examined how demographic and health-related characteristics of Spanish-dominant (SD), Bilingual (BIL), and English-dominant (ED) Latino men and women aged 25–64 differed among members of a large Northern California health plan.

**Methods:**

This observational study was based on data from cohorts of 171 SD (requiring an interpreter), 181 BIL, and 734 ED Latinos aged 25–64 who responded to random sample health plan member surveys conducted 2005–2006. Language groups were compared separately by gender on education, income, behavioral health risks (smoking, obesity, exercise frequency, dietary practices, health beliefs), health status (overall health and emotional health, diabetes, hypertension, high cholesterol, heartburn/acid reflux, back pain, depression), computer and Internet access, and health education modality preferences.

**Results:**

Compared with ED Latinos, higher percentages of the SD and BIL groups had very low educational attainment and low income. While groups were similar in prevalence of diabetes, hypertension, and high cholesterol, SD were less likely than ED Latinos to rate overall health and emotional well-being as good, very good, or excellent and more likely to report heartburn and back pain (women only). The groups were similar with regard to smoking and obesity, but among women, SD were more likely to be physically inactive than ED, and BIL were less likely than SD and ED groups to eat <3 servings of fruit/vegetables per day. SD and BIL of both genders were significantly less likely than ED Latinos to believe that health practices had a large impact on health. Compared to ED men and women, SD and BIL Latinos had significantly lower Internet and computer access. As a result, SD Latinos had a greater preference for lower technology health education modalities such as videos and taped phone messages.

**Conclusion:**

There are important differences among Latinos of different English language proficiency with regard to education, income, health status, health behaviors, IT access, and health education modality preferences that ought to be considered when planning and implementing health programs for this growing segment of the U.S. population.

## Background

Between 1990 and 2000, the U.S. Hispanic population increased from 22.3 million to 35.3 million, and by 2050, it is expected that over 97 million Hispanics will live in the United States, accounting for nearly one-fourth of the U.S. population [1,2]. In the Western United States, the Hispanic presence is much larger (in 2005, Hispanics, primarily originating from Mexico and Central America, comprised 35% of the adult population in California) [3]. Hispanics are a diverse group, varying significantly with regard to country of origin, demographic factors, health-related characteristics and acculturation level including generation in the U.S., and dominant language. In general, Hispanics of Mexican and Central American descent (hereafter referred to as Latinos) are significantly more disadvantaged on many socioeconomic and health measures than those of Cuban, Puerto Rican, or South American descent [4].

Among Latinos, being born in the US tends to be positively associated with higher educational attainment, higher income, English proficiency [5] and Internet access [6], but negatively associated with health-promoting behaviors and chronic health problems [4]. As a result of generational differences, the overall demographic and health characteristics of the Latino population are likely to change substantially as the percentage of the Latino population that is born in the U.S. or immigrate at a very young age increases. The 2002 National Survey of Latinos found that nearly all Latinos born in the U.S. speak English, with 46% of second generation and 22% of third-plus generations being bilingual, compared with only 28% of foreign-born Latinos (24% of whom are bilingual) [5]. Educational attainment is also significantly higher among second and third generation Latinos as compared with foreign-born Latinos, with 75% vs. 46%, respectively, completing at least high school and 14% vs. 9% having college degrees. While Latino adults are significantly less likely than non-Hispanic Whites and African-Americans to use the Internet, English-dominant (prefers to communicate in English) and bilingual Latinos are approximately three times more likely to use the Internet than Spanish-dominant (very limited or no English language proficiency) Latinos, and the difference between English-dominant Latinos and non-Hispanic Whites Latinos significantly diminishes after adjusting for education [6].

Health behaviors and chronic diseases have also been shown to be subject to the effects of generational differences due to acculturation. Based on evidence of several studies of the effects of acculturation on Latino health behaviors and health, Lara *et al *posit that the effect of acculturation, while not absolute, is generally negative with regard to health behaviors such as dietary practices, smoking, obesity, and physical inactivity, resulting in an increasing rate of diabetes and onset at younger ages [7]. For example, while studies have found dietary changes that are healthy (e.g., decreased use of lard, cream, and sausage) and unhealthy (e.g., less fresh fruits and vegetables, rice, beans, and more sweets) among more acculturated versus less acculturated Latina women, the researchers judged the overall effect of acculturation on diet to be more negative than positive [7-9]. The effect of acculturation on Latino smoking appears to be gender-dependent; acculturation is associated with increased smoking prevalence among women, but has little or no effect on men. Ultimately, this results in the more acculturated men and women having smoking rates similar to those among non-Hispanic Whites [7,10,11]. Three studies found a higher prevalence of obesity among more acculturated Latino adults and adolescents than among the less acculturated [12-14]. In contrast to these negative effects, Crespo *et al*. found that acculturation was positively associated with participation in leisure-time physical activity [15].

According to the 2005 California Health Interview Survey, there were approximately 900,000 Latinos of Mexican or Central American descent aged 20–64 covered by non-Medicaid health insurance in California in 2005, approximately 23% of all insured adults in this age group [3]. Of these, approximately 24% had limited ability to speak English, 51% spoke English well or very well, and 25% spoke only English. The objective of our study was to explore the heterogeneity of Spanish-dominant, bilingual, and English-dominant Latinos aged 25–64 who were members of a large Northern California health plan with regard to educational attainment, income, health-related characteristics, access to information technology, and preferred methods for receiving health information and health education. Our intent is to provide information to health plans serving a culturally diverse Latino population with information that might aid in understanding differences in health care needs and outcomes for these populations, as well as for planning health education services.

## Methods

### Study design and populations

The Kaiser Permanente of Northern California (KPNC) Adult Member Health Survey, conducted every three years starting in 1993, is a project designed to inform policy makers, researchers, administrators, and clinicians both inside and outside the Health Plan about healthcare-related characteristics and preferences of insured adults [16]. This confidential survey, conducted with an age, gender, and geographically stratified random sample of 42,000 KPNC adult health plan members aged 20 and over, covers sociodemographic characteristics, health status and health conditions, health-related behaviors and lifestyle risk factors, use of selected medications and dietary supplements, preventive services, and previous use of and interest in different methods of obtaining health education. While in 2005 the survey was primarily conducted using a mailed questionnaire, the survey could also be completed on a secure website or by phone. Due to cost constraints, the survey has only been conducted in English, and people known to require a translator or require written information in a language other than English have been excluded from the sample. However, since the numbers of members who speak little or no English have been increasing over recent years, and since learning about health and health care disparities has become a priority for not only the health plan, but for society at large, the decision was made to conduct a pilot survey in Spanish for Latino members with limited or no English proficiency (Spanish-dominant group). Both surveys were approved by KPNC's Institutional Review Board.

The English-dominant Latino group was comprised of 532 women and 388 men aged 25–64 who had self-identified as being of Mexican-American or Central American ethnicity on the 2005 KPNC Member Health Survey (MHS). Of these 920 Latino members, 93 women and 87 men who indicated that they preferred to use Spanish when talking about or learning about their health were classified as bilingual; the remaining 433 women and 301 men were classified as English-dominant. Since the Health Plan did not have computerized information about race-ethnicity, country of birth, or generation in the United States for all members, it was not possible to estimate what percentages of bilingual and English-dominant Latinos responded to the survey.

The Spanish Member Health Survey questionnaire was a slightly modified version of the 2005 Member Health Survey, translated into Spanish using terms and wording chosen to be understandable to a monolingual Mexican-American or Central American adult with limited education. The survey sample included an age- and gender stratified random sample of 309 adults aged 25–64 who were identified from a health plan patient demographics database as requiring a Spanish-language interpreter for clinic visits and Spanish-language preference for written materials (at the time, information about language preference and interpreter needs was available for 95% of KPNC members). A survey packet consisting of a cover letter, information sheet, self-administered questionnaire, and return envelope was sent in May 2006, with the offer of a $10 gift card for participating in the survey via phone interview or self-administered questionnaire. Nonrespondents were called approximately 3 weeks after the initial mailing, at which time the bilingual research assistant encouraged them to complete the survey with her over the phone or offered to send another copy of the self-administered questionnaire if that was their preference. Those who were sent a second copy of the questionnaire but did not mail back a completed form were contacted one final time to offer the phone interview. In all, 78 women and 93 men (66.7% and 57.3%, respectively, of people who were contacted by mail or phone) completed the survey, 126 by self-administered and 45 by interviewer-administered questionnaire over the phone. Of the 171 respondents in the Spanish-dominant group, 69.8% (n = 120) self-identified on the survey as Mexican-American, 16.3% (n = 28) as Central American, and 13.9% (n = 24) as Other Hispanic/Latino.

### Study variables

Sociodemographic measures included age, education (highest level of school completed), and total household income from all sources in 2004, before taxes. Country of origin was not ascertained because we had been advised that including this question would reduce the number of recent immigrants and non-documented workers willing to participate. Both the education and income questions had categorical responses. Based on past research, a greater number of response categories for very low levels of educational attainment were included in the Spanish language survey.

Health status was assessed using the standard question of "In general, would you say your health is excellent, very good, good, fair, or poor." Follow-up questions asked for separate ratings of physical health (including pain) and emotional/mental using the same scale. For the Spanish survey, the rating categories were "Muy bueno, bueno, regular, malo." Respondents were also asked to indicate from a checklist whether during the past 12 months, they had or had taken medication for a number of health conditions, including diabetes, high blood pressure, high cholesterol, frequent heartburn or acid reflux, severe back pain or sciatica, depression, sadness, or very low spirits lasting at least 2 weeks, and frequent problems with sleep.

Health behaviors were ascertained using several questions. Current smoking status was derived from two questions that asked whether the individual had ever regularly smoked cigarettes, and if so, whether the individual smoked now, even occasionally. Obesity (BMI ≥ 30 Kg/m^2^) was derived from self-reported height and weight. Exercise frequency was based on response to a question about how often the individual usually got physical exercise, such as walking, swimming, gardening, golf, and tennis. People who indicated "3 to 4 times a week" or "5 or more times a week" were considered to be exercising ≥ 3 times a week. People who indicated an exercise frequency of less than 1 to 2 times a week (i.e., "2 to 4 times a month", "once a month or less," or "never") were considered to get exercise less than once a week (sedentary behavior). Dietary questions focused more on behaviors than evaluating content of the diet. Usual number of servings of fruits and vegetables was based on a single item "During an average day, about how many servings of fruits and vegetables do you usually eat (1 serving = a half cup or a medium piece)." This single item fits with the public health message to consume "5 A Day," not differentiating between fruits and vegetables. DiSogra and Hudes previously showed that using two items that asked about average number of servings for total fruit and total vegetables separately was a good way to estimate the fruit and vegetable consumption to obtain population estimates for tracking purposes, although it would likely produce a slightly higher estimate than estimates based on multiple items about servings of specific foods [17]. Dietary behavior related to fat intake was assessed by asking about how often the individual tried to eat reduced fat (low fat or nonfat) foods. People who indicated doing this "all of the time" or "most of the time" were considered to usually try to eat reduced fat foods. Daily multivitamin use was taken from a checklist of dietary and herbal supplements that the individual had used during the past 12 months. Belief about the relationship of behavioral risks and health was based on response to a question "How much do you think habits/lifestyle, such as exercise, what you eat, and your weight, can affect your health." People who indicated "quite a bit" or "extremely" were considered to believe that these had a large effect, while those who indicated "not at all" or "a little bit" were considered to believe that these had little or no effect.

Access to information technology (personal computer, Internet) was ascertained by the questions "Do you have access to a personal computer?" and "Do you have access to the Internet?" with response options of "Yes, at home," "Yes, at another location," or "No" [access]. Use of selected health education modalities in the prior 12 months and interest in future use of different health education modalities were assessed by two checklist questions, the latter of which read, "In addition to talking with your doctor, how would you prefer to learn about taking care of health problems and improving your health?" The exact wording and format of questions in English and Spanish is available upon request from the first author.

### Analysis

All analyses were done using weighted data. The Bilingual and English-dominant respondents had all previously been assigned post-stratification weights so that analyses using weighted data would reflect the age (in 5-year intervals), gender, and geographic composition (KPNC service populations) of the full adult membership at the time of the survey. The Spanish-dominant sample was similarly assigned weights based on age (5-year intervals) and gender of Spanish-dominant members aged 25–64 at the time of the survey. Because previous research has shown significant gender differences in population-based sociodemographic characteristics and behavioral health risks, analyses for men and women were performed separately.

Analyses were performed using weighted data and SAS (Statistical Analysis Software) procedures for analysis of data obtained from complex survey designs [18]. Proc Surveyfreq was used to estimate the unadjusted percentages and confidence intervals (see Additional file 1). Because the linguistic groups differed by age (Table 1), and age was associated with many of the health-related behaviors and health indicators, the weighted percentage estimates for all three linguistic groups were then age-adjusted to the age distribution of the English-dominant Latinos (women ages 25–39: 0.516, ages 40–64: 0.484; men ages 25–39: 0.523, ages 40–64: 0.477) using Proc Surveyreg as outlined by Gossett et al. [19]. To assess whether the age-adjusted percentages significantly differed between pairs (e.g., English-dominant women vs. Bilingual women), Proc Surveyreg was also used to subtract one age-adjusted estimate from a second and apply a t-test for difference [19]. Finally, Proc Surveylogistic models were used to confirm the Proc Surveyreg results and evaluate whether controlling for education in addition to age reduced differences between the linguistic groups found after adjusting for age alone.

**Table 1:** Age distributions of Spanish-dominant, Bilingual, and English-dominant Latino study groups prior to age-adjusting

	**Women**			**Men**		
		
**Age**	**Spanish-dominant ** (n = 78)	**Bilingual ** (n = 99)	**English-dominant ** (n = 433)	**Spanish-dominant ** (n = 93)	**Bilingual ** (n = 87)	**English-dominant ** (n = 301)
25–39 yr	53.2%	43.3	51.6	56.9	60.4	52.3
40–64 yr	46.8%	56.7	48.4	43.0	39.6	47.7
Mean (SE)	40.2 (1.0)	42.3 (1.0)	40.3 (0.5)	39.4 (1.2)	39.0 (0.8)	40.3 (0.6)
Median	39	42	38	37	38	39

## Results

### Sociodemographic characteristics

Latinos significantly differed across language preference groups with regard to educational attainment and income (Table 2). Both Spanish-dominant and bilingual Latinos were significantly more likely than English-dominant Latinos to have less than 12 years of formal education and were significantly less likely to be college graduates. In addition, the educational attainment of the Spanish-dominant group was significantly lower than that of the bilingual group. Among the Spanish-dominant group, 35% of men and women had only attended primary school and 24% (32% of men and 16% of women) had attended only through middle school, while among the Bilingual group, 16% of men and 6% of women had < 9 years of formal education (not shown in table). Approximately 31% of Spanish-dominant women and 12% of Spanish-dominant men had any post-secondary education, as compared with 49% of women and 44% of men in the bilingual group. With regard to household income, compared with English-dominant men and women, significantly higher percentages of Spanish-dominant men and women and bilingual men reported a household income of ≤ $25,000, and significantly lower percentages of men and women in both groups reported a household income greater than $50,000. While differences in the income extremes remained statistically significant after adjusting for age, they were somewhat diminished when educational attainment was taken into account. However, Spanish-dominant men and women were significantly more likely to be at the lowest income level and significantly less likely to be at the higher income levels than their bilingual counterparts, even after controlling for the effects of age and education.

**Table 2:** Age-adjusted socioeconomic status (SES) characteristics of Spanish-dominant, Bilingual, and English-dominant Latinos Aged 25–64 Years

	**Women**			**Men**		
		
**SES Characteristics**	**Spanish-dominant ** (n = 78)% (se)	**Bilingual ** (n = 99)% (se)	**English-dominant ** (n = 433)% (se)	**Spanish-dominant ** (n = 93)% (se)	**Bilingual ** (n = 87)% (se)	**English-dominant ** (n = 301)% (se)
**Post-Secondary Education**						
No						
< 12 yrs formal school	59.5 (6.8) ****, ^d^	25.9 (4.8) ****	6.5 (1.4)	81.6 (4.2) ****, ^d^	25.8 (5.5) ***	5.9 (1.4)
High school graduate	9.4	24.8	14.7	5.9	30.1	23.0
Yes	31.1 (6.5) ****, ^a^	49.3 (5.6) ****	78.8 (2.1)	12.5 (3.6) ****, ^d^	44.1 (6.0) ****	71.1 (3.0)
Some college/tech school	16.7	33.2	48.2	5.7	32.6	39.4
College graduate	14.4 (5.4) **	16.1 (4.1) **	30.6 (2.3)	6.8 (2.9)****	11.5 (3.2) ****	31.7 (3.0)
						
**Household Income**						
≤ $25,000	33.7 (6.0) ****, ^a^	15.7 (4.3)	8.9 (1.4)	33.6 (6.7) ****, ^b^	12.7 (4.1)	5.0 (1.6)
$25,001–35,000	33.4	17.5	5.6	40.9	13.9	7.0
$35,001–50,000	18.1	18.4	18.5	13.6	26.2	19.2
≥ $50,000	14.7 (5.4) ****, ^d^	48.4 (5.7) **	66.9 (2.5)	11.9 (3.5) ****, ^b^	47.2 (6.2) **	68.8 (3.2)
$50,001–65,000	5.4	15.9	16.1	5.8	18.8	11.1
$65,001–80,000	3.5	20.6	15.3	6.1	11.0	19.4
> $80,000	5.9 (4.4) ****	11.9 (3.8) ****	35.6 (2.5)	< 0.1 (--)****, ^d^	17.4 (4.5) ****	38.3 (3.1)

* p < 05; ** p < .01; *** p < 001; ****p < .0001 by t-test, Spanish-dominant and Bilingual compared to English-dominant.

^a ^p < 05; ^b ^p < .01; ^c ^p < 001; ^d ^p < .0001 by t-test, Spanish-dominant compared to Bilingual

### Health status and behavioral/lifestyle risk factors

Spanish-dominant Latinos were significantly less likely than bilingual and English-dominant Latinos to rate their overall health as being good, very good, or excellent, with the majority considering their health to be "regular" (the Spanish equivalent of "fair") (Table 3). Bilingual and English-dominant Latinos did not significantly differ with regard to overall health status, and very few people in any of the groups rated their health as poor. Spanish-dominant were also significantly less likely than bilingual and English-dominant Latinos to rate their mental/emotional health as good, very good, or excellent. There were no statistically significant differences across groups with regard to prevalence of diabetes, high blood pressure, or high cholesterol, although Spanish-dominant men and women had significantly higher prevalence of heartburn/acid reflux than bilingual and English-dominant men and women. In addition, Spanish-dominant women were significantly more likely than the other groups of women to report back pain and more likely to report feeling depressed or in very low spirits, although the difference was not statistically significant. All statistically significant differences remained significant after adjusting for education.

**Table 3:** Age-adjusted health status and behavioral health risk characteristics of Spanish-dominant, Bilingual, and English-dominant Latinos Aged 25–64 Years

	**Women**			**Men**		
		
**Health Characteristics**	**Spanish-** **dominant ** (n = 78) % (se)	**Bilingual ** (n = 99) % (se)	**English-** **dominant** (n = 433) % (se)	**Spanish-** **dominant ** (n = 93) % (se)	**Bilingual ** (n = 87) % (se)	**English-** **dominant ** (n = 301) % (se)
**Health Status**						
Self-reported overall health						
Very Good/Excellent	14.5 (3.6) ****, ^d^	39.5 (5.3) ****	48.9 (2.6)	23.7 (5.9) ****, ^b^	50.0 (6.1)	53.2 (3.3)
≥ Good	42.6 (6.3) ****	90.4 (2.8)	90.5 (1.5)	50.2 (6.4) ****, ^d^	87.2 (4.7)	89.2 (2.1)
Fair/"Regular"^1^	56.9 (6.4) ****, ^d^	9.6 (2.8)	8.2 (1.5)	46.4 (6.4) ****, ^d^	12.8 (4.7)	9.7 (2.0)
Self-reported mental and emotional health						
Very Good/Excellent	34.6 (7.1) **, ^a^	56.9 (5.7)	54.3 (2.6)	30.1 (6.5) ****, ^d^	63.9 (6.2)	61.3 (3.2)
≥ Good	67.9 (6.4) *, ^a^	86.3 (3.8)	84.1 (1.9)	76.7 (5.8) *	90.2 (4.8)	90.1 (1.9)
Diabetes	10.6 (4.0)	13.1 (4.0)	8.1 (1.4)	11.8 (4.0)	13.7 (4.0)	11.2 (1.8)
High Blood Pressure	17.3 (4.9)	14.4 (3.3)	14.3 (1.7)	14.5 (4.0)	16.3 (4.1)	19.1 (2.2)
High Cholesterol	12.7 (4.2)	5.8 (2.1)	9.0 (1.4)	18.6 (3.9)	16.7 (4.2)	14.7 (1.9)
Heartburn or acid reflux	27.5 (6.2) **, ^a^	10.9 (3.5)	10.3 (1.6)	25.4 (5.4) **, ^b^	6.7 (2.8)	8.3 (1.9)
Back Pain	31.3 (6.5) **, ^b^	9.0 (3.1)	9.6 (1.6)	17.9 (5.0)	9.3 (3.1)	15.5 (2.5)
Depression	21.1 (5.5)	10.9 (3.6)	13.3 (1.7)	9.1 (2.9)	6.1 (2.9)	10.5 (2.0)
						
**Behavioral Health Risks**						
Current Smoker	5.4 (2.4)	2.6 (1.9) *	7.4 (1.3)	15.5 (6.4)	8.3 (3.7)	10.1 (1.9)
Overweight (BMI ≥ 25 kg/m^2^)	67.7 (6.8)	67.4 (5.4)	67.3 (2.5)	81.5 (5.6)	84.3 (4.4)	81.9 (2.3)
Obese (BMI ≥ 30 kg/m^2^)	28.4 (6.5)	33.5 (5.5)	37.4 (2.6)	20.1 (5.4) **	27.5 (5.8)	37.8 (3.3)
Exercises < once/week	45.3 (7.2) **, ^a^	23.4 (5.1)	22.2 (2.2)	32.0 (6.1)	19.9 (5.0)	20.8 (2.7)
Exercises ≥ 3 times/week	31.4 (6.7) *	39.9 (5.5)	46.7 (2.6)	37.3 (7.1) *	35.5 (6.0)	56.8 (3.3)
Usually tries to eat reduced fat foods	42.9 (6.8)	33.5 (5.1)	33.8 (2.5)	40.2 (6.5)	30.9 (5.4)	32.1 (3.1)
Usually eats ≥ 3 servings fruit/vegetables per day	43.2 (7.3)^a^	25.4 (4.5) **	40.5 (2.5)	34.0 (6.5)	28.3 (5.6)	21.8 (2.7)
Takes a daily multivitamin	19.2 (4.7) ****, ^a^	34.3 (5.3)	45.9 (2.6)	22.3 (5.8) *	35.5 (5.8)	35.9 (3.2)
Belief about relationship of behavior/lifestyle and health						
Have large effect	51.4 (6.9) ****	58.2 (5.5) ****	88.3 (1.7)	35.3 (6.3) ****	39.9 (5.9) ****	84.3 (2.4)
Have little or no effect	36.4 (6.7) ****	27.0 (4.9) ****	4.0 (1.0)	51.2 (6.5) ****	35.1 (5.8) ****	7.5 (1.9)

^1 ^On English form, "Fair " was between Good and Poor; on Spanish form, "Regular" was between "Bueno" and Malo".

* p < 05; ** p < .01; *** p < 001; ****p < .0001 by t-test, Spanish-dominant and Bilingual compared to English-dominant.

^a ^p < 05; ^b ^p < .01; ^c ^p < 001; ^d ^p < .0001 by t-test, Spanish-dominant compared to Bilingual

The three language groups did not differ on current smoking, being overweight, and usually trying to eat reduced fat foods (Table 3). However, Spanish-dominant women were significantly more likely than the other two groups of women to get exercise less than once a week, and Spanish-dominant men and women were significantly less likely than English-dominant men and women to get exercise ≥ 3 times a week. Bilingual women were significantly less likely than both Spanish-dominant and English-dominant Latino women to eat at least three servings of fruits and vegetables per day, and both Spanish-dominant and bilingual women were significantly less likely than English-dominant women to take daily multivitamins. Both Spanish-dominant and bilingual Latinos were also significantly less likely than their English-dominant counterparts to believe that factors such as diet, exercise, and weight, could have a big impact on their health and significantly more likely to believe these factors had little or no impact on their health. All differences that were statistically significant before adjusting for age remained significant after adjusting for age. However, after the additional adjustment for education, Spanish-dominant Latinas were significantly more likely than English-dominant women to try to limit high fat foods, and Spanish-dominant men were significantly less likely than English-dominant men to eat ≥ 3 servings of fruit/vegetables per day (data not shown).

### Access to computer and the Internet

Among both men and women, Spanish-dominant and bilingual Latinos were significantly less likely than English-dominant Latinos to have access to a personal computer and to the Internet from any location (Table 4), with the Spanish-dominant Latino group having significantly less access than the bilingual Latino group. All group-level differences that were statistically significant after adjusting for age remained significant after controlling for education and household income < $35,000 (not shown), although adjusting for education and income diminished the disparities between the English-dominant men and the bilingual Spanish-dominant men.

**Table 4:** Age-adjusted comparisons of computer and Internet access and preferred methods for obtaining health education, Spanish-dominant, Bilingual, and English-dominant Latinos Aged 25–64

	**Women**			**Men**		
		
**Internet Access and Health Education Preferences**	**Spanish-** **dominant ** (n = 78) % (se)	**Bilingual ** (n = 99) % (se)	**English-** **dominant ** (n = 433) % (se)	**Spanish-** **dominant ** (n = 93) % (se)	**Bilingual ** (n = 87) % (se)	**English-** **dominant ** (n = 301) % (se)
**Personal Computer**						
At home	50.2 (6.9) ****	65.0 (5.2) **	80.7 (2.0)	34.3 (5.7) ****, ^c^	64.7 (5.7) **	82.7 (2.6)
At home/other location	58.3 (6.6) ****	73.4 (4.3) ****	90.8 (1.3)	37.8 (6.1) ****, ^d^	70.0 (5.5) ***	90.2 (1.9)
**Internet**						
At home	39.3 (6.9) ****, ^a^	56.8 (5.5) ***	77.5 (2.0)	25.5 (4.5) ****, ^d^	58.7 (5.9) ***	81.4 (2.6)
At home/other location	44.9 (6.9) ****, ^b^	69.1 (4.6) ****	88.9 (1.4)	30.7 (5.7) ****, ^d^	66.8 (5.8) ***	89.7 (2.0)
						
**Information Source Used in Past 12 months**						
Health education handouts	14.6 (4.3)	14.3 (3.8)	18.8 (2.1)	11.1 (3.2)	4.9 (2.4) **	14.1 (2.2)
Health handbook	37.7 (6.6)	36.9 (5.4)	32.0 (2.4)	21.2 (5.4)	17.2 (4.4)	24.3 (2.9)
Health phone messages	13.8 (5.3) *, ^a^	0.8 (0.8)	2.6 (0.1)	3.6 (2.0)	2.4 (2.3)	1.2 (0.6)
Information from a website	9.5 (3.9)	2.0 (1.7) ****	17.1 (1.9)	0.3 (--) ****	2.8 (1.6) ****	17.1 (2.5)
Health education programs	5.5 (2.5) ***	2.7 (3.8) **	16.2 (2.0)	6.5 (3.3)	7.7 (3.6)	8.1 (1.6)
						
**Preferred Methods**						
Small group appointments with a clinician/educator	29.4 (6.4)^a^	12.9 (4.0)	17.8 (20.0)	30.9 (6.1) **, ^a^	14.4 (4.7)	10.7 (1.8)
Individual counseling	33.4 (6.2)	39.9 (5.3)	41.6 (2.5)	34.7 (6.0)	25.3 (5.0) **	42.7 (3.2)
Brief telephone counseling	26.3 (5.1)^b^	9.4 (3.1) **	19.6 (2.1)	36.7 (6.6) **, ^d^	48.4 (2.8) *	16.7 (2.5)
1/2- full day workshop	20.4 (5.4) *, ^a^	7.1 (3.6)	7.4 (1.3)	12.5 (4.1)	10.2 (3.8)	8.4 (1.8)
Multi-session program						
Traditional class	10.8 (4.7)	7.1 (2.8)	8.7 (1.5)	4.0 (2.2)	7.4 (3.0)	5.5 (1.2)
Group program by phone	4.5 (2.2)	2.7 (1.6)	1.6 (0.6)	7.4 (3.5) *, ^a^	0 (0)	1.4 (0.6)
Internet-based	2.3 (1.6) *	8.5 (3.5)	7.2 (1.3)	7.4 (3.3)	3.6 (2.2)	8.8 (2.0)
Information from a website	11.0 (4.5) **	7.3 (3.1) ****	25.3 (2.2)	12.8 (4.1) **	8.4 (3.1) ****	28.7 (3.0)
Computer program	10.7 (3.3)	8.6 (3.0)	13.7 (1.7)	15.1 (4.3)	10.4 (3.4)	17.5 (2.6)
Health phone messages	21.5 (4.8) **, ^b^	3.5 (2.1)	5.2 (1.1)	19.0 (5.5) **, ^a^	4.8 (2.3)	4.1 (1.3)
Health videos	44.9 (6.5) ****, ^a^	21.5 (4.5)	16.6 (1.9)	57.2 (6.6) ****, ^d^	22.4 (5.1)	19.8 (2.5)
Health programs on TV	49.5 (6.9) ****, ^d^	18.0 (3.8)	18.9 (2.0)	29.3 (5.1)	25.4 (5.3)	22.3 (2.7)
Health newsletters	56.2 (6.7) *, ^c^	24.6 (4.9) **	39.8 (2.5)	54.2 (6.7) **, ^c^	22.9 (5.3)	30.9 (3.1)
Short handouts or articles	59.7 (6.6)^b^	31.7 (5.3) *	46.5 (2.6)	46.3 (6.6)	30.5 (5.6)	36.7 (3.1)

* p < 05; ** p < .01; *** p < 001; ****p < .0001 by t-test, Spanish-dominant and Bilingual compared to English-dominant.

^a ^p < 05; ^b ^p < .01; ^c ^p < 001; ^d ^p < .0001 by t-test, Spanish-dominant compared to Bilingual

#### Health education preferences

Given the relatively low levels of personal computer and Internet access, both Spanish-dominant and bilingual Latinos were significantly less likely than English-dominant Latinos to have sought health information from the Internet during the previous 12 months and their preferred modalities did not require computer or Internet use (Table 4). However, the differences between English-dominant and Spanish-dominant men and women diminished after adjusting for education (not shown). Spanish-dominant Latinas were significantly more likely than bilingual and English-dominant Latinas to have obtained information via dial-up health phone tapes, and both Spanish-dominant Latino women and men were significantly more likely than the other two groups to indicate health phone as a preferred method for health education. Spanish-dominant Latinos were also significantly more likely than both bilingual and English-dominant Latinos to express interest in health newsletters, health videos, health programs on TV (women only), and small group appointments, but did not differ from the bilingual and English-dominant groups in showing little interest in multi-session programs such as traditional classes, phone-based classes, and Internet-based programs. Bilingual Latinos were significantly less interested than English-dominant Latinos in brief health-related counseling in person (men only) or by phone (men and women), perhaps because they assumed counseling would occur in English.

## Discussion

Our comparison (in the context of a health care plan) of Spanish-dominant, bilingual, and English-dominant Latino men and women aged 25–64 revealed several differences that have implications for the provision of health education services. While the linguistic groups did not differ with regard to prevalence of diabetes, high blood pressure, or high cholesterol, the Spanish-dominant Latinos had higher percentages of men and women reporting heartburn and women reporting back pain, suggesting that Spanish language educational materials for prevention and management of these health problems might help improve quality of life for this population segment. Similar to findings of other studies [7], Spanish-dominant Latinos were also significantly less likely than the bilingual and English-dominant Latinos to consider their health to be good, even after adjusting for education. Our relatively small Spanish-dominant Latino sample did not allow us to assess whether the lower rating of health reflects actual differences in health and well-being or, alternatively, cultural differences in how people think about health status. Spanish-dominant and bilingual Latinos were also significantly less likely than English-dominant counterparts to believe that health risk factors such as diet, exercise, and weight had a large impact on health, partly due to low level of formal education and less prior exposure to the concept of risk reduction in their home countries. Thus, they may be less ready to act on the general messages being given by health care providers and the public health community about eating more fruits and vegetables, trying to reduce the amount of fat in their diet, and increasing frequency of exercise to improve health.

With regard to behavioral health and lifestyle factors, our results confirm the findings of Crespo *et al *that Spanish-dominant Latinos are more likely to report being physically inactive than English-dominant Latinos [15]. Moreover, their hypothesis that Spanish-dominant Latinos may get substantial amounts of physical activity during work or activities of daily living (which would not have been captured by our exercise question) is indirectly supported by our finding that Spanish-dominant Latinos were less likely than English-dominant Latinos to be obese.

In our sample, a very large proportion of Spanish-dominant Latinos expressed an interest in receiving health education. Because Spanish-dominant Latinos had much lower levels of formal education, household income, and access to personal computers and the Internet than English-dominant Latinos, it was not surprising to find greater preference among those speaking only Spanish for lower technology modalities of health education, including videos, television programs, and taped health messages accessible by phone, and lower preference for Internet-based sources. Despite the lower levels of education, more than one-fourth of the Spanish-dominant Latino group indicated that they had gotten information from the health handbook they received from the health plan and more than half were interested in receiving health newsletters and short articles or brochures about health. This suggests that while providing Spanish-language materials and programs via the Internet may have great logistical and economic appeal, these modalities will likely not reach a majority of Spanish speaking Latinos who require and are eager to receive health education. Moreover, because of the low household income, it is likely that even those with Internet access from home are not using very high speed DSL or broadband connections nor computers with very fast processing speed and large RAM, making it very difficult for them to interact with websites that have extensive graphics, to interact with online programs, and to download materials.

This study has both strengths and limitations. While the fact that the data all came from a health plan population in Northern California might affect the generalizability of the results to the broader insured or uninsured population, this can also be viewed as a strength in that it provides a more controlled environment for observing differences associated with dominant language. All of the survey respondents were not only insured for primary care, but being seen by primary care clinicians whose practice guidelines calls for screening for and counseling their patients about behavioral/lifestyle health risks and making referrals to the health plan's health education resources. Limitations of the study include its use of non-validated self-report data; the relatively small numbers of Spanish-dominant and bilingual Latinos; likely under-participation in the English language survey by bilingual and English-dominant Latinos of very low educational attainment and relatively low overall response to that survey; lack of information about country of birth and generation in the United States; and inability to weight the bilingual and English-dominant Latinos data to the age-gender distribution of Latino health plan members with some English proficiency because census-type race/ethnicity statistics for the membership were not available.

## Conclusion

Our findings highlight important differences among Latinos of different English language proficiency with regard to educational attainment, income, health status, health behavior, technology access, and health education modality preferences that ought to be considered when planning and implementing health programs for this growing segment of the U.S. population. Specifically, there is a great need to provide Spanish-dominant and bilingual Latinos with education about the relationship between current health habits/lifestyle and future health and functional status in order to sustain the favorable health status indicators (lower morbidity/mortality rates, lower prevalence of many chronic diseases, lower rates of disability) that have been well-documented among Spanish-dominant or lesser acculturated Latino populations in the United States [4,7]. For the Spanish-dominant population, education about depression and back pain targeted to women and heartburn/acid reflux targeted to both men and women might provide a teachable moment to explore this relationship between lifestyle choices and health outcomes. Finally, Latinos with limited English proficiency appear to be very interested in obtaining health education, but it will likely be more accessible if it is provided through non-Internet-based modalities such as videos, taped telephone messages, Spanish language print materials, and small group visits with a doctor or patient educator. Further research should be done to determine the generalizability of these results to other U.S. Latino populations and to monitor whether the identified differences in education, income, health behaviors, health issues, and preferred health education modalities diminish or widen over time.

## Competing interests

The authors declare that they have no competing interests.

## Authors' contributions

NG conceived, designed, and conducted the surveys, performed the statistical analyses, interpreted the data, and drafted the manuscript. CI consulted on the data interpretation and helped to draft the manuscript. Both authors read and approved the final manuscript.

## Pre-publication history

The pre-publication history for this paper can be accessed here:



## Supplementary Material

Additional file 1 Tables A1-A3 with percentages unadjusted for age. Description: This file contains the same comparisons of the 3 Latino samples (Spanish-dominant, bilingual, and English-dominant) found in Tables 1, 2, and 3 based on weighted respondent data that was not further age-adjusted to a standard population to make the age distributions of all the groups comparable. Click here for file
