# Supplementary material for: Health-related characteristics and preferred methods of receiving health education according to dominant language among Latinos Aged 25 to 64 in a large Northern California health plan
Source: BMC Public Health. 2008 Sep 9;8:305. doi: 10.1186/1471-2458-8-305 (PMC2556675; doi:10.1186/1471-2458-8-305)
Supplement: Additional file 1 — Tables A1-A3 with percentages unadjusted for age. Description: This file contains the same comparisons of the 3 Latino samples (Spanish-dominant, bilingual, and English-dominant) found in Tables 1, 2, and 3 based on weighted respondent data that was not further age-adjusted to a standard population to make the age distributions of all the groups comparable. [file 1471-2458-8-305-S1.doc]

**Table 2A. Socioeconomic status (SES) characteristics of Spanish-dominant, Bilingual, and**

**English-dominant Latinos aged 25-64 years, unadjusted for age**

| **SES Characteristics** | **Women** | | |  | **Men** | | |
| --- | --- | --- | --- | --- | --- | --- | --- |
|  | **Spanish-dominant**  **(n=78)**  **%** | **Bilingual**  **(n=99)**  **%** | **English-dominant**  **(n=433)**  **%** |  | **Spanish-dominant**  **(n=93)**  **%** | **Bilingual**  **(n= 87)**  **%** | **English-dominant**  **(n=301)**  **%** |
| **Age** |  |  |  |  |  |  |  |
| 25-39 yr | 53.2 | 43.3 | 51.6 |  | 56.9 | 60.4 | 52.3 |
| 40-64 yr | 46.8 | 56.7 | 48.4 |  | 43.0 | 39.6 | 47.7 |
| Mean (SE) yr | 40.2 (1.0) | 42.3 (1.0) | 40.3 (0.5) |  | 39.4 (1.2) | 39.0 (0.8) | 40.3 (0.6) |
| Median yr | 39 | 42 | 38 |  | 37 | 38 | 39 |
|  |  |  |  |  |  |  |  |
| **Post-secondary education** |  |  |  |  |  |  |  |
| No | 69.3 | 51.8 | 21.2 |  | 88.2 | 56.0 | 28.9 |
| <12 yrs formal school | 59.7 | 26.7 | 6.5 |  | 82.6 | 24.7 | 5.9 |
| High school graduate | 9.6 | 25.1 | 14.7 |  | 5.6 | 31.3 | 23.0 |
| Yes | 30.7 ****^,c^ | 48.2 **** | 78.8 |  | 11.8 ****^,d^ | 44.0 **** | 71.1 |
| Some college/tech school | 16.4 | 31.7 | 48.2 |  | 5.4 | 32.9 | 39.4 |
| College graduate | 14.3 * | 16.5 ** | 30.6 |  | 6.4 **** | 11.1 **** | 31.7 |
|  |  |  |  |  |  |  |  |
| **Household Income** |  |  |  |  |  |  |  |
| ≤ $25,000 | 33.8 ****^,a^ | 16.0 | 8.9 |  | 33.8 ****^,a^ | 13.6 * | 5.0 |
| $25,001-35,000 | 33.5 | 18.1 | 5.6 |  | 41.6 | 13.5 | 7.0 |
| $35,001-50,000 | 18.1 | 18.3 | 18.5 |  | 13.0 | 25.4 | 19.2 |
| ≥ $50,000 | 14.6 ****^,b^ | 47.6 ** | 66.9 |  | 11.5 ****^,b^ | 47.6 ** | 68.7 |
| $50,001-65,000 | 5.3 | 16.1 | 16.1 |  | 5.5 | 21.1 | 11.1 |
| $65,001-80,000 | 3.4 | 19.7 | 15.3 |  | 6.0 | 10.8 | 19.4 |
| > $80,000 | 5.9 *** | 11.8 **** | 35.6 |  | <0.1 ****^,d^ | 15.6 *** | 38.2 |

* p<05; ** p<.01; *** p<001; ****p<.0001 by Rao-Scott chi-square, Spanish-dominant and Bilingual compared to

English-dominant

^a^ p<05; ^b^ p<.01; ^c^ p<001; ^d^ p<.0001 by Rao-Scott chi-square, Spanish-dominant compared to Bilingual

**Table 3A. Health Status and behavioral health risks of Spanish-dominant, Bilingual and**

**English-dominant Latinos aged 25-64 years , unadjusted for age**

|  | **Women** | | |  | **Men** | | |
| --- | --- | --- | --- | --- | --- | --- | --- |
|  | **Spanish-dominant**  **(n=78)**  **%** | **Bilingual**  **(n=99)**  **%** | **English-dominant**  **(n=433)**  **%** |  | **Spanish-dominant**  **(n=93)**  **%** | **Bilingual**  **(n= 87)**  **%** | **English-dominant**  **(n=301)**  **%** |
| **Health Status** |  |  |  |  |  |  |  |
| Self-reported overall health |  |  |  |  |  |  |  |
| Very Good/ Excellent | 14.8 ****^,c^ | 40.7 | 48.9 |  | 27.5 **^,a^ | 49.7 | 53.6 |
| ≥ Good | 43.5 ****^,d^ | 89.5 | 90.6 |  | 51.2****^,c^ | 87.6 | 89.3 |
| Fair / ”Regular” ^1^ | 57.7 ****^,d^ | 10.4 | 8.2 |  | 43.8 ****^,c^ | 12.4 | 9.6 |
| Self-reported mental and  emotional health |  |  |  |  |  |  |  |
| Very Good/Excellent | 34.6 *^,a^ | 57.3 | 54.3 |  | 30.4 ***^,b^ | 63.3 | 61.3 |
| Good | 33.3 | 29.2 | 29.8 |  | 46.9 * | 26.7 | 28.8 |
| Diabetes | 10.3 | 14.3 | 8.1 |  | 11.4 | 11.7 | 11.2 |
| High Blood Pressure | 16.9 | 16.4 | 14.3 |  | 14.2 | 15.0 | 19.1 |
| High Cholesterol | 12.4 | 6.8 | 9.0 |  | 17.3 | 15.1 | 14.7 |
| Heartburn or acid reflux | 27.3 ***^,b^ | 10.6 | 10.3 |  | 25.3 ***^,c^ | 6.1 | 8.3 |
| Back Pain | 31.1 **** ^,c^ | 8.9 | 9.6 |  | 17.4 | 9.9 | 15.5 |
| Depression | 20.9 | 11.1 | 13.4 |  | 8.4 | 6.4 | 10.5 |
|  |  |  |  |  |  |  |  |
| **Behavioral Health Risks** |  |  |  |  |  |  |  |
| Current Smoker | 5.4 | 2.5 | 7.4 |  | 15.4 | 7.4 | 10.1 |
| Overweight (BMI ≥ 25 Kg/m^2^) | 67.7 | 69.3 | 67.6 |  | 81.8 | 83.1 | 81.9 |
| Obese (BMI ≥ 30 kg/m^2^) | 28.3 | 34.1 | 37.6 |  | 19.7 * | 26.3 | 37.8 |
| Exercises < once/week | 45.2 ****^,c^ | 22.4 | 22.2 |  | 31.4 | 19.6 | 20.9 |
| Exercises ≥ 3 times/week | 31.4 * | 40.8 | 46.7 |  | 37.3 * | 54.7 | 56.8 |
| Usually tries to eat  reduced fat foods | 42.6 | 34.2 | 33.8 |  | 40.1 | 29.7 | 32.1 |
| Usually eats ≥ 3 servings  fruit/vegetables per day | 43.1^,a^ | 26.4 * | 40.5 |  | 34.4 | 28.6 | 21.8 |
| Takes a daily multivitamin | 19.3 **** | 33.3 * | 45.9 |  | 22.2 | 35.9 | 35.9 |
| Belief about relationship of behavioral risks and health |  |  |  |  |  |  |  |
| Have large effect | 51.4 **** | 56.7 **** | 88.3 |  | 34.1 **** | 39.0 **** | 84.3 |
| Have little or no effect | 36.4 **** | 28.0 **** | 4.0 |  | 52.8 **** | 35.6 **** | 7.5 |

^1^ On English form, “Fair “ was between Good and Poor; on Spanish form, “Regular” was between “Bueno” and “Malo”

* p<05; ** p<.01; *** p<001; ****p<.0001 by Rao-Scott chi-square, Spanish-dominant and Bilingual compared to

English-dominant.

^a^ p<05; ^b^ p<.01; ^c^ p<001; ^d^ p<.0001 by Rao-Scott chi-square, Spanish-dominant compared to Bilingual

**Table 4A. Computer and internet Access and preferred methods for obtaining health education,**

**Spanish-dominant, Bilingual, and English-dominant Latinos Aged 25-64, unadjusted for age**

| **Internet Access and Health Education Preferences** | **Women** | | |  | **Men** | | |
| --- | --- | --- | --- | --- | --- | --- | --- |
|  | **Spanish-dominant**  **(n=78)**  **%** | **Bilingual**  **(n=99)**  **%** | **English-dominant**  **(n=433)**  **%** |  | **Spanish-dominant**  **(n=93)**  **%** | **Bilingual**  **(n= 87)**  **%** | **English-dominant**  **(n=301)**  **%** |
| **Personal Computer** |  |  |  |  |  |  |  |
| At home | 50.1 **** | 63.3 *** | 80.7 |  | 33.3 ****^,b^ | 63.4 **** | 82.7 |
| At home/other location | 58.4 **** | 71.0 **** | 90.8 |  | 37.0 ****^,c^ | 69.0 ** | 90.2 |
| **Internet** |  |  |  |  |  |  |  |
| At home | 39.4 **** | 55.9 **** | 77.5 |  | 24.4 ****^,d^ | 57.6 *** | 81.3 |
| At home/other location | 44.9 ****^,a^ | 66.9 **** | 88.9 |  | 30.0 ****^,d^ | 66.2 **** | 89.7 |
|  |  |  |  |  |  |  |  |
| **Information Source Used in Past 12 months** |  |  |  |  |  |  |  |
| Health education handouts |  |  |  |  |  |  |  |
| Health handbook | 37.4 | 32.3 | 32.0 |  | 21.1 | 18.9 | 24.3 |
| Health phone messages | 13.8 ***^,c^ | 1.0 | 2.6 |  | 3.4 | 1.1 | 1.3 |
| Information from a website | 9.7^,a^ | 2.0 **** | 17.1 |  | 0.3 ****^,a^ | 3.4 **** | 17.1 |
| Health education programs |  |  |  |  |  |  |  |
|  |  |  |  |  |  |  |  |
| **Preferred Methods** |  |  |  |  |  |  |  |
| Small group appointments with a clinician/educator | 29.4 * | 12.1 | 17.8 |  | 30.5 ***^,a^ | 12.6 | 10.7 |
| Individual counseling | 33.6 | 36.4 | 41.6 |  | 33.9 | 28.7 ** | 42.7 |
| Brief telephone counseling | 26.6 | 10.1 *** | 19.6 |  | 36.9 **^,d^ | 11.5 * | 16.7 |
| ½- full day workshop | 20.4 ** | 5.1 | 7.4 |  | 12.1 | 9.2 | 8.4 |
| Multi-session program |  |  |  |  |  |  |  |
| Traditional class | 10.8 | 8.1 | 8.7 |  | 3.6 | 8.0 | 5.5 |
| Group program by phone | 4.6 | 3.0 | 1.6 |  | 7.2 * | 0.0 | 1.4 |
| Internet-based | 2.3 | 7.1 | 7.2 |  | 6.6 | 3.4 | 8.8 |
| Information from a website | 11.0 * | 6.1 *** | 25.3 |  | 12.2 ** | 9.2 **** | 28.7 |
| Computer program | 10.9 | 9.1 | 13.7 |  | 14.9 | 10.3 | 17.5 |
| Health phone messages | 21.8 ****^,c^ | 3.0 | 5.2 |  | 18.7 ***^,a^ | 5.7 | 4.1 |
| Health videos | 45.1 ****^,b^ | 24.2 | 16.6 |  | 56.7 **** | 23.0 | 19.8 |
| Health programs on TV | 49.5 ****^,d^ | 22.2 | 18.9 |  | 28.1 | 24.1 | 22.3 |
| Health newsletters | 56.1 *^,c^ | 25.3 *** | 39.8 |  | 53.7 **^,b^ | 23.0 | 30.9 |
| Short handouts or articles | 59.7^,c^ | 32.3 * | 46.5 |  | 45.9 | 31.0 | 36.7 |

* p<05; ** p<.01; *** p<001; **** p<.0001 by Rao-Scott chi-square, Spanish-dominant and Bilingual compared to

English-dominant

^a^ p<05; ^b^ p<.01; ^c^ p<001; ^d^ p<.0001 by Rao-Scott chi-square, Spanish-dominant compared to Bilingual
